# Antimicrobial activity of *Bacillus* sp. isolated strains of wild honey

**DOI:** 10.1186/s12906-022-03551-y

**Published:** 2022-03-19

**Authors:** Somayeh Hallaj-Nezhadi, Rasoul Hamdipour, Mohamad Shahrvirani, Roya Zare tin, Florence Chapeland-leclerc, Gwenael Ruprich-Robert, Solmaz Esnaashari, Babak Elyasi Far, Azita Dilmaghani

**Affiliations:** 1 Drug Applied Research Center, Tabriz University of Medical Sciences, Tabriz, Iran; 2 Department of Drug &Food Control, Faculty of Pharmacy, Tabriz University of Medical Sciences, Tabriz, Iran; 3 Department of Pharmaceutical Biotechnology, Faculty of Pharmacy, Tabriz University of Medical Sciences, Tabriz, Iran; 4 Institut Des Energies de Demain (IED), UMR 8236, Univ Paris Descartes, Sorbonne Paris Cité, F-75205 Paris, France; 5 Biotechnology Research Center, Tabriz University of Medical Sciences, Tabriz, Iran; 6 Department of Physiology and Pharmacology, School of Medicine, Dezful University of Medical Sciences, Dezful, Iran

**Keywords:** *Bacillus* sp., Antimicrobial activity, Human pathogens, Phytopathogenic pathogenes, GC–MS

## Abstract

**Background:**

Multi-drug resistant bacteria hazards to the health of humans could be an agent in the destruction of human generation. Natural products of *Bacillus* species are the main source to access progressive antibiotics that can be a good candidate for the discovery of novel antibiotics. Wild honey as a valuable food has been used in medicine with antimicrobial effects.

**Objective:**

*Bacillus* strains isolated from wild honey were evaluated for the potential antimicrobial activity against human and plant bacterial and fungal pathogens.

**Methods:**

Three bacterial isolates were identified as strain Khuz-1 (98.27% similarity with *Bacillus safensis* subsp. Safensis strain FO-36b^T^), strain Khuz-2 (99.18% similarity with *Bacillus rugosus* strain SPB7^T^), and strain Khuz-3 (99.78% similarity with *Bacillus velezensis* strain CR-502^ T^) by 16S rRNA gene sequences. The strains were characterized by their ability to inhibit the growth of human and phytopathogenic fungi.

**Results:**

The results indicated that *B. rugosus* strain Khuz-2 inhibited the growth of phytopathogenic and human fungal more effective than other ones. It seems that the strain Khuz-2 has a suitable antimicrobial and antifungal potential as a good candidate for further pharmaceutical research.

**Conclusion:**

Based on the results of GC–MS, Pyrrolo [1,2-a] pyrazine-1,4-dion, hexahydro-3-(2-methylpropyle) (PPDHM) was the major compound for all strains which have a various pharmacological effect. Isolation and identification of beneficial bacteria from natural sources can play an important role in future pharmaceutical and industrial applications.

**Supplementary Information:**

The online version contains supplementary material available at 10.1186/s12906-022-03551-y.

## Introduction

Honey as a valuable food has been used in ancient medicine. It has a remarkable effect on the remediation of wound healing, bedsore treatment, and ulcers [1]. Recently, the progression of multi-drug resistant bacteria hazards to the health of humans which could be an agent in the destruction of human generation. Natural products of bacteria are the main source to access progressive antibiotics that can be a good candidate for the discovery of novel antibiotics [2]. However, the finding of new antibiotics is very scarce at the industrial level and needs to examine by new methods [3]. In 1966 Burkholder et al. extracted a pyrrole antibiotic from marine bacteria for the first time [4]. Consequently, various antimicrobial compounds were introduced and produced by different micro-biome, such as archaea, bacteria and fungi [5]. It seems that the isolation and identification of novel bacteria can be a new approach in the achievement of unknown natural sources which leads us to find new antibiotics for the accumulation of our starved pharmaceutical business [6]. Natural products discovery efforts began in pharmaceutical companies, mainly in the United States, Europe and Japan, and modest efforts fol-lowed in isolated academic laboratories worldwide [7]. Since 2013, around 1453 new chemical entities have been accepted by US Food and Drug administration [7]. The Most important natural product used in the anti-infective area, especially anti- bacterial therapy. Around twelve natural product is used to treat Gram-positive or Gram-negative bacterial infections in humans and animals [8]. In this research, the antimicrobial ability of *Bacillus* sp. strains were discussed which was isolated and identified for the first time from wild honey collected from Khuzestan Province, Iran.

## Materials and methods

### Sample collection and growth conditions

A literature search was directed up to 2018, on the electronic databases of Scopus, PubMed, and Web of Science. The search was accomplished by using the following search strings in the title/abstract/keywords: “Antimicrobial Activity of environmental bacteria” AND “Wild Honey*” OR both of them. Obtained articles were imported to EndNoteX9 reference management software. All articles were separately screened for, duplicity, and eligibility by two authors individually [9]. According to our search, it seemed that there is few research about the investigation of the bacterial population in Khuzestan wild honey and the Identification of these bacteria can be helpful in both basic and applied research. Therefore, for this aim, honey samples were collected from Shushtar city, Khuzestan Province, Iran. Khuzestan Province as the historical Iranian province is situated in the southwest of Iran, in the neighborhood of the Persian Gulf and the Iran-Iraq border (32°02′44″N 48°51′24″E). The samples were transported to the laboratory in sterile condition. The microbial population of samples was enriched using specific media [10] and the colonies were isolated from 18 to 24 h. Isolates were cultured in the nutrient broth, containing (g/L), NaCl 9, MgSO_4_.7H_2_O 9.7, MgCl_2_.H_2_O 7.0, CaCl_2_ 3.6, KCl 2.0, NaHCO_3_ 0.06 and NaBr 0.026, where pH was adjusted to 7.3 ± 0.2 before autoclaving. Cultures were incubated at 30 °C in an orbital shaker, at 150 rpm min^−1^ for 72 h. To culture on solid media, 12–15 gl^−1^ agar was added to the new nutrient broth, then it was incubated at 30 °C for 48 h. Cell morphology and biochemical tests were carried out to identify isolates [6, 11].

### Physiological characteristics

The principal tests used for strains Khuzestan1 (Khuz-1), Khuzestan2 (Khuz-2) and Khuzestan3 (Khuz-3). These purposes are Hydrogen Sulphide Production (H_2_S), potassium hydroxide (KOH), Urease Test(URE), Catalase Test (CAT), Tween20,40 and 80 test, starch hydrolysis test, Gelatin hydrolysis test, L-Tyrosine test [12, 13].

Bacterial isolates were tested for growth at different pH (4- 11), (using increments of 1 pH unit) on lab made nutrient broth. The pH values were adjusted using buffer system including 0.1 M citric acid/0.1 M sodium citrate for pH 4.0–5.0; 0.1 M KH_2_PO_4_/0.1 M NaOH for pH 6.0–8.0; 0.1 M NaHCO_3_/0.1 M Na_2_CO_3_ for pH 9.0–10.0; 0.05 M and Na_2_HPO_4_/0.1 M NaOH for pH 11.0 with different temperature ranges (5, 10, 20, 30, 40 and 50 °C). Samples were measured by UV absorbance at 600 nm wave length (OD: 600) after 48 h.

Following identification, susceptibility test of the isolated bacteria was performed using the disk diffusion sensitivity method employing paper disks impregnated with seventeen different types of antibiotics (Padtan Teb Co., Iran): cefalexin (30 μg/mL^−1^), chloramphenicol (30 μg/mL^−1^), azithromycin (15 μg/mL^−1^), amikacin (30 μg/mL^−1^), ampicillin (10 μg/mL-1), penicillin (10 μg/mL^−1^), rifampicin (5 μg/mL^−1^), erythromycin (15 μg/mL^−1^), ciprofloxacin (5 μg/mL^−1^), cefoxitin (30 μg/mL^−1^), ceftriaxone (30 μg/mL^−1^), nitrofurantoin (300 μg/mL^−1^), doxycycline (5 μg/mL^−1^), tetracycline (30 μg/mL^−1^), amoxicillin (10 μg/mL^−1^), gentamicin (10 μg/mL^−1^) and erythromycin (15 μg/mL^−1^). Each bacterium spread over the surface of NA medium in petri dishes followed by the distribution of paper disks impregnated with the antibiotics. Then, the cultures remained under incubation at 30 °C.

### Molecular Identification

Genomic DNA extraction was done by DNA extraction Kit (Cinagene DNa plus, South Korea), according to manufacturer’s protocol. Universal used primers were 16F 5’- AGAGTTTGATCCTGGCTCAG-3’, and reverse 16R; 5’- TACCTTGTTAGGACTTCACC-3’ primers [14]. Each amplification reaction contained 1µL of each primer, dNTP (10 mM) 0.5 µm, PCR buffer 2.5 µL, MgCl_2_ (50 mM) 0.75 µL, template DNA 1 µL, smartaq DNA polymerase 0.2 µL, and dH_2_O 19.05 µL, in a final volume of 25µL. The 16S rRNA gene amplification protocol was 95 °C for 5 min, followed by 40 cycles of 95 °C for 1 min, 55 °C for 55 s and 72 °C for 1 min and 25 s, with final 10 min extension at 72 °C [15]. High-Pure PCR Product Purification kit (Roche) was used for the amplified fragment purification and then sequenced by utilizing forward and reverse primers by Macrogen (Korea) [16]. The phylogenic relationship of the isolates was determined by comparing the sequencing data with the related 16S rRNA gene sequences in the GenBank database of the National Center for Biotechnology Information (https://www.ncbi.nlm.nih.gov/), via BLAST search. In addition, the sequences were conducted in the EzBioCloud identity service led to retrieving its closest phylogenetic neighbors [17]. The 16S rRNA gene reads were assembled using Chromas pro software and aligned using the multiple sequence alignment program CLUSTAL_X (version 1.83) [18] Phylogenetic analysis was performed using the neighbor-joining method in MEGA version 7 software package [14, 19].

### Preparation of pathogenic microorganisms

The antimicrobial properties of isolates were assayed by the agar well diffusion method. Based on the previous studies, we selected the serious pathogenic microorganisms that are involved in globally important human or plant diseases [20, 21]. In this study, *Bacillus cereus* ATCC 11,778, *Escherichia coli* O157 PTCC 1276, *Klebsiella pneumonia* PTCC 10,031, *Shigella flexneri* PTCC 1234, *Pseudomonas aeroginosa* ATCC 10,231, *Streptococcus mutams* ATCC 35,668 and *Candida albicans* ATCC 10,231 were studied as human pathogenic organisms and *Fusarum oxysparum (*IBRC-M 30,067*), Aspergillus flavus (*IBRC-M 30,029*), Neurospora crassa (*IBRC-M 30,138*), Botrytis cinerea* (IBRC-M 30,162*), Erwinia amylovora* (IBRC-M 10,748), *Psudomonas syringea pv. Syringae* (IBRC-M 10,702*)*, *Xanthomonas campestris* (IBRC-M 10,644) and, *Rhizobium radiobacter* (IBRC-M 10,701) were used as plant pathogenic. According to the agar well diffusion method, described by Ennahar et al*.,* 2000 the bacterial extracts were filtered through a 0.22 µm pore filter membrane after centrifugation of bacterial culture at 5000 rpm for 10 min. The pathogenic microorganisms (10^7^ CFU/mL) were inoculated into a sterile plate with 20 mL of their selective media. Subsequently, the plate was gently shacked for even spread and suitable mixing of the microorganisms and media. It was permitted to solidify. Then, 5 wells of about 6 mm in diameter were prepared on the surface of the agar plates using a sterile cylinder and the plates were turned upside down. The wells were labeled with a marker. Each well was occupied with 0.1 mL of the bacterial extracts. Then, the plates were incubated at 37 °C for 24 h and the inhibition zone was measured. Finally, the results were organized [22].

### Antimicrobial assays

First, each colony of bacteria was cultured in 50 mL of liquid nutrient medium and incubated at 30 °C with shaking for 72 h. Then, to separate the extract from the medium, the culture was centrifuged at 4000 rpm for 20 min. Subsequently, the supernatant was collected and filtered through a 0.22 µm membrane filter [6, 23].

### Minimum inhibitory concentration (MIC) and Minimum bactericidal concentration (MBC)

The MIC of the extract against bacteria was determined using the micro broth dilution method as recommended by the National Committee for Clinical Laboratory Standards (NCCLS). The MIC value is considered as the lowest concentration that completely inhibited bacterial growth after 48 h of incubation at 30 °C. The various concentrations (10^–1^ g / ml to 10^–8^ g / ml) of the extract were used to detect the MIC value against tested pathogens. 16 μl suitable medium was added into each well and 20 μl of the 0.5 McFarland suspension of pathogen bacterial (1:10 diluted) was added into the wells. Finally, different concentrations of the extract (20 μl) was added into the same wells and sterile broth was added into the wells to makeup to 300 μL. The positive control wells contain 0.5 McFarlend (OD: 600) of the pathogen and culture medium and the negative control wells contained an antimicrobial extract and culture media. After cultivation, the microplates were mixed wells to make the mixture quite uniform and placed in an incubator for 24 h at 30° C. The MIC value was determined by the first well in which the visible growth of microorganisms was inhibited. For the determination of MBC, a portion of liquid (5 μl) from each well that did not exhibit any growth was taken and then incubated at 30 oC for 24 h. The lowest concentration that revealed no visible bacterial growth after sub-culturing was taken as MBC [6].

### Minimum inhibitory concentration (MIC) and (MFC)

To determine the MIC value for bacterial extract against fungal pathogens, the macro broth dilution method was used. In brief, serial dilutions of bacterial extract ranging from 10–1 g/ml to 10–8 g/ml were prepared in 96 well microtitration plates [24]. Then, 100 µL suitable medium was added to the well and 100 µL of fungal inoculums at a concentration of 10^6^ CFU/mL. This serial dilution was repeated and the positive control (medium with fungal) and culture medium and the negative control (medium only) contained an antimicrobial extract and culture media. the plate was incubated at a suitable temperature. After 48 h, tubular opacity and growth of fungi were evaluated in comparison with the control plate. To obtain the MFC, 10 ml of each dilution was taken from each well and spread on suitable medium. Plates were incubated at 28 ℃ for 72 h. The MFC was defined as the lowest concentration capable of inhibiting 99% fungal growth or fewer colonies [23, 24].

### Gas Chromatography–Mass Spectroscopyanalysis (GC–MS)

GC/MS analysis as a suitable method for identification of unknown and different substances within a test sample was carried out using Gas Hewlett PackardHP-5890 series II equipped with split/splitless injector and a capillary column (30 m, 0.25 mm, 0.25 lm) fused with phenylpolysilphenylene siloxane. The injector and detector temperatures were set at 280 and 300 °C, respectively, and the oven temperature was kept at 80 °C for 1 min, rose to 300°Cat20°C/min. Helium was used as carrier gas at a constant flow of 1.0 ml/min. A volume of 2ll was injected in the splitless mode and the purge time was 1 min. The MS (Hewlett–Pack-ard 5889B MS Engine) with selected ion monitoring (SIM) was used. The mass spectrometer was operated at 70 eV and scan fragments from 50 to 650 m/z. Peak identification of crude algal extracts was performed based on comparing the obtained mass spectra with those available in NIST library [25].

### Statistical analysis

Statistical analysis was performed by using SPSS software version 16 to calculate the mean of zones of inhibition on tested microorganisms.

## Result

### Isolation and Identification of the *Bacillus* Strain

**Fig. 1:**
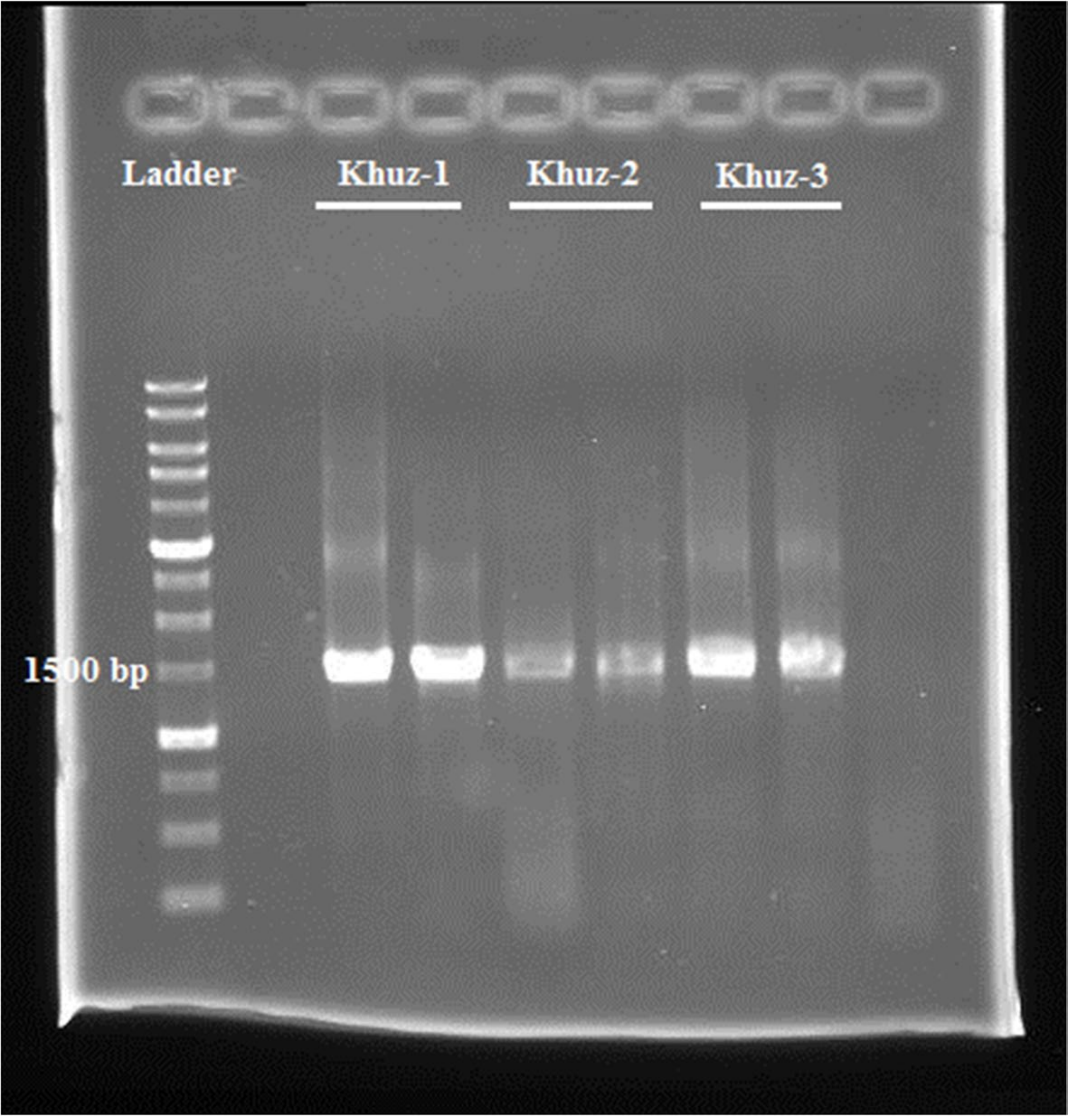
The gel electrophoresis of 16S rRNA gene sequences of isolated strains. The length of fragments were approximately ≈ 1500 bp

**Fig. 2:**
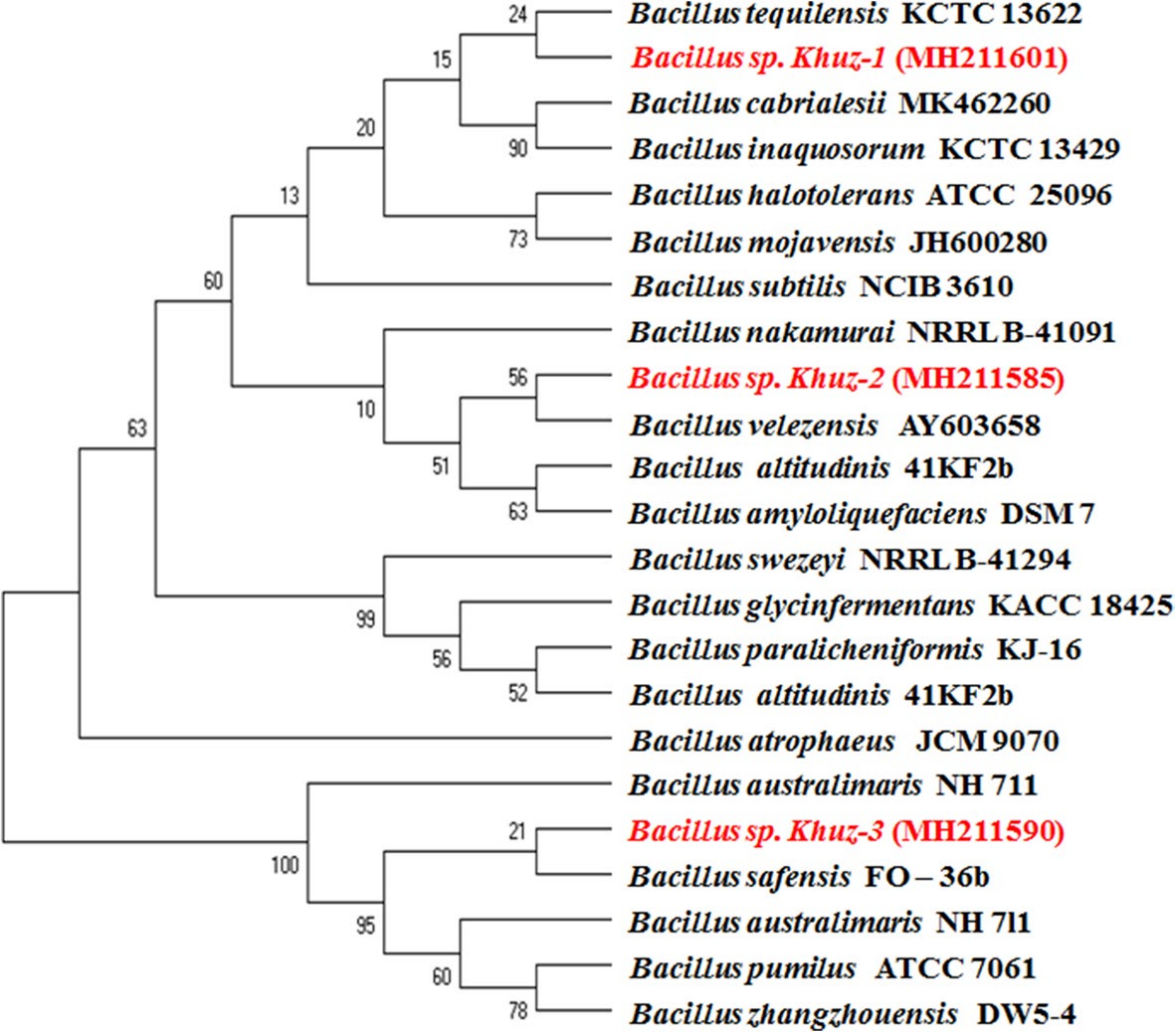
Neighbor-joining phylogenetic tree [26] derived from 16S rRNA gene sequence data [14] showing the positions of seven isolated bacteria including strains Khuz-1, Khuz-2 and Khuz-3 among related bacteria. Numbers at branch points are bootstrap percentages based on 500 replicates. Bar, 0.02 changes per nucleotide position [19]

In the present study, three different *Bacillus* strains were isolated from Shushtar city, Khuzestan Province from wild honey, which is named Khuz- 1, 2 and 3 (abbreviation of Khuzestan). The isolates were purified on NA medium and preserved in a refrigerator maintained at-70 °C [16]. We doubted that the bacteria isolated belonged to genus *Bacillus*. To confirm this hypothesis, we isolated DNA for the usual PCR of the 16S rRNA gene. For this target, we amplified a 1.5 kb DNA sub-fragment of the 16S rRNA gene, which is extremely maintained within the genus *Bacillus* (Fig. 1). The 16S rRNA gene sequences of mentioned strains Khuz- 1, 2 and 3 were submitted in gene bank with accession numbers; MH211590, MH211601 and MH211585, respectively. In addition, the phylogenetic tree of 16S rRNA genome sequencing of the isolates showed that they are closely related to *Bacillus safensis, Bacillus rugosus, Bacillus velezensis* (Fig. 2).

### Biochemical characterization of bacteria

Bacteria were identified based on the colony, pigment and microscopy shape. All strains were selected for further assays and their biochemical characteristics are summarized in Table 1.

**Table 1:** The isolated strains and their biochemical characteristics

ID	KoH	H_2_O_2_	Tween20	Tween40	Tween80	Starch	Gelatin	Tyrosine	Urease	H_2_S
Strain Khuz- 1	+	+	+	+	-	-	-	+	+	** + **
Strain Khuz- 2	+	+	+	+	+	+	+	+	+	** + **
Strain Khuz- 3	+	+	+	+	-	+	+	+	+	** + **

+ Positive reaction,—negative reaction

All isolates were Gram-positive. The growth was observed in the range of pH 4 to 10 with the ability to grow up to 10 pH. The optimum pH for all strains were 7 and 8 (Table 2). Additionally, all bacteria can grow the widespread range of temperature at 5 to 50 ^0^C. The optimum temperature for all strains was between 20 to 30 ^0^C, which is recorded in Table 2. As shown in Table 3, the antibiotic resistance of isolated bacteria towards different antibiotics was also determined for phenotypical characterization of isolates. According to our results, all strains were highly resistant to Cefazolin and Cefalexin.

**Table 2:** The range of temperature (°C) and pH of isolated strains and their optimized ranges

ID									
Temperature °C	**5**	**10**	**15**	**20**	**30**	**40**	**50**	**55**	**60**
Strain Khuz-1	-	+	+	+ +	+	+	+	-	**-**
Strain Khuz-2	-	+	+	+ +	+	+	+	-	**-**
Strain Khuz-3	-	+	+	+ +	+	+	+	-	**-**
pH	**4**	**5**	**6**	**7**	**8**	**9**	**10**	**11**	**12**
Strain Khuz-1	-	+	+	+ +	+	+	+	+	**-**
Strain Khuz-2	-	+	+	+ +	+	+	+	+	**-**
Strain Khuz-3	-	+	+	+ +	+	+	+	+	**-**

+ Positive reaction, +  + strongly positive reaction,—negative reaction

**Table 3:** Culture susceptibility test of the identified bacterial contaminants to different antibiotics. Data are expressed as the average of three determinations ± standard deviations. *NI* Not Inhibited

Property	Strain Khuz-1	Strain Khuz-2	Strain Khuz-3
Azithromycin	26 ± 0.4 s	19 ± 0.2 s	20 ± 0.5 s
Rifampicin	17 ± 0.4 s	27 ± 0.2 s	3 ± 0.2r
Erythromycin	26 ± 0.2 s	26 ± 0.1 s	25 ± 0.3 s
Amikacin	32 ± 0.4 s	26 ± 0.3 s	21 ± 0.2 s
Ciprofloxacin	32 ± 0.2 s	32 ± 0.1 s	31 ± 0.3 s
Tetracyclin	25 ± 0.1 s	37 ± 0.4 s	15 ± 0.2 s
Cefaxitin	26 ± 0.3 s	32 ± 0.4 s	29 ± 0.3 s
Gentamicin	24 ± 0.1 s	25 ± 0.2 s	26 ± 0.3 s
Cefalexin	40 ± 0.4 s	39 ± 0.1 s	38 ± 0.4 s
Ampicilin	31 ± 0.2 s	30 ± 0.1 s	26 ± 0.3 s
Chloramphenicol	5 ± 0.2r	25 ± 0.3 s	29 ± 0.2 s
Ceferiaxon	17 ± 0.4 s	36 ± 0.2 s	31 ± 0.3 s
Nitrofurntion	22 ± 0.3 s	26 ± 0.3 s	20 ± 0.1 s
Cefazolin	42 ± 0.2 s	39 ± 0.1 s	40 ± 0.3 s
Pencillin	36 ± 0.1 s	34 ± 0.2 s	27 ± 0.3 s
Amoxicillin	33 ± 0.2 s	31 ± 0.3 s	25 ± 0.3 s
Doxycycline	31 ± 0.3 s	27 ± 0.1 s	26 ± 0.2 s

### Antimicrobial and Antifungal activity of bacteria

These two isolates (*Bacillus safensis* strain Khuz-1 and *Bacillus velezensis* strain Khuz-3) were able to produce inhibition zones against the pathogenic microorganisms. On the other hand, all of the isolates were not active against human bacterial pathogens like *Bacillus cereus, Echerchia coli, Klesbsiella pneumonia, Shigella aureus* and *streptococcus aureus*.

Our results indicated that *Bacillus rugosus* strain Khuz-2 (MH211601) have the ability to produce inhibition zones against human pathogen, *C. albicans* (21 mm) and depicted antimicrobial effect against plant pathogen *F. oxysparum, N. crassa, B. cinerea,, E. amylovora. R. radiobacter* and *A. flavus*. However, *B. rugosus* wasn’t active against *X. campestris* and *P. syringe*. This isolate showed the highest inhibition zone against *E. amylovora* (30 mm) (Table 4, 5). *B. velezensis* was not active against human pathogen; unlike being active against *F.oxysparum* and *N. crassa* (20 and 16 mm). On the other hand, *B. safensis* wasn’t active against any pathogen. The MIC and MBC values showed that *B. velezensis* extract against Plants microbial display antimicrobial activity just against *N. crassa* and *F. oxysparum*. The activity of *B. velezensis* against *N. crassa* was considerable in the highest values (MIC = 25 μg/ml). However, the extraction of *B. velezensis* biomass did not show any activity against human and the others pathogens (Table 6). The activity of *B. rugosus* against plant and human tested fungi were considerable, with the highest values for *R. radiobacter* (MIC = 50 μg/ml). The extract was inactive against human bacteria. (Table 7).

**Table 4:** Inhibition zones (mm) against different human pathogenic microorganisms. Data are expressed as the average of three determinations ± standard deviations. *NI* Not Inhibited

Human pathogen	Strain Khuz-1	Strain Khuz-2	Strain Khuz-3
*Candida albicans*	NI	21	21

**Table 5:** Inhibition zones (mm) against different plant pathogens microorganisms

Plant Pathogens ^a^	Strain Khuz-1	Strain Khuz-2	Strain Khuz-3
*Psudomonas syringe pv. Syringae*	NI	NI	NI
*Xanthomonas campestris*	NI	NI	NI
*Erwinia amylovora*	NI	30	NI
*Rhizobium radiobacter*	NI	21	NI
*Fusarum oxysparum*	NI	19	20
*Aspergillus flavus*	NI	12	NI
*Neurospora crassa*	NI	15	16
*Botrytis cinerea*	NI	20	NI

^a^Values are mean of three different tests. Means followed by the same letters are not significantly different in each row for each isolates (*p* < 0.05),—> 100 μg/ml

**Table 6:** minimum inhibitory concentration (MIC) and minimum fungicidal concentration (MFC) *B. velezensis* extract against Plants microbial of the most effective (values in μg/ml)

Pathogens^a^	MIC	MFC
*Fusarum oxysparum*	12.5 μg/ml	12.5 μg/ml
*Neurospora crassa*	25 μg/ml	25 μg/ml

^a^Values are mean of three different tests. Means followed by the same letters are not significantly different in each row for each isolates (*p* < 0.05),—> 100 μg/ml

**Table 7:** minimum inhibitory concentration (MIC) and minimum fungicidal concentration (MFC) *B. rugosus* extract against Human and Plants microbial of the most effective (values in μg/ml)

Pathogens^a^	MIC	MBC
*Erwinia amylovora*	25 μg/ml	25 μg/ml
*Rhizobium radiobacter*	50 μg/ml	50 μg/ml
*Fusarum oxysparum*	12.5 μg/ml	12.5 μg/ml
*Aspergillus flavus*	25 μg/ml	25 μg/ml
*Neurospora crassa*	25 μg/ml	25 μg/ml
*Botrytis cinerea*	25 μg/ml	25 μg/ml
*Candida albicans*	25 μg/ml	25 μg/ml

^a^Values are mean of three different tests. Means followed by the same letters are not significantly different in each row for each isolates (*p* < 0.05),—> 100 μg/ml

### Chemical composition of extracts

A gas chromatography-mass spectrometry (GC–MS) analysis of the extract was used for the identification of the components [25]. Following the GC–MS analysis, the identified compounds with their percentages, formula, retention time and molecular weight have been represented in Table 8, 9 and 10 for three strains. The data were available in supplementary PDF S1, 2 and 3 files.

**Table 8:** Chemical composition of Khuz-1 strain using GC/MS analysis

No	Compound name	Percentage	Rt ^a^	Formula	Molecular weight
1	methylcyclohexane	3.69	11.65	C7H14	98
2	2-Piperidinone	3.37	20.65	C5H9NO	99
3	Acetamide, 2-amino-N-(1-methylethyl)	4.81	20.93	c5h12N2O	116
4	Acetamidoacetic acid-Glycine, N-acetyl	2.40	24.35	C4H7NO3	117
5	Decane-2-methyl	1.12	25.46	C11H24	156
6	1- Propanamine-2-methyl	13.30	29.42	C4H11N	73
7	Phenol, 2,4-bis(1, 1-dimethylethyl)	1.60	31.30	c14h22O	206
8	Heptadecane	1.44	31.61	C17H36	240
9	1-Octanamine	8.17	32.31	C8H19N	129
10	Piperidine, 3-methyl	1.76	34.99	C6H13N	99
11	Octadecane	0.80	37.01	C18H38	254
12	2- Decene, 3-methyl- (Z)	9.94	37.82	c11h22	154
13	2- Decene, 3-methyl- (Z)	1.92	38.31	c11H22	154
14	Pyrrolo[1,2-a] pyrazine-1,4-dion,hexahydro-3-(2-methylpropyle)	5.29	40.01	C11H18N2O2	210
15	Pyrrolo[1,2-a] pyrazine-1,4-dion,hexahydro-3-(2-methylpropyle)	20.51	40.46	C11H18N2O2	210
16	Pyrrolo[1,2-a] pyrazine-1,4-dion,hexahydro-3-(2-methylpropyle)	5.77	40.62	C11H18N2O2	210
17	Pyrrolo[1,2-a] pyrazine-1,4-dion,hexahydro-3-(2-methylpropyle)	2.56	40.71	C11H18N2O2	210
18	Ergotaman-3'.6'.18-trione, 9,10-dihydro-12'hydroxy-2'-methyl-5'-(phenylmethyl)-,(5'.alpha.,10 alpha)	1.76	48.20	C33H37N5O5	583
19	Pyrrolo[1,2-a] pyrazine-1,4-dion,hexahydro-3-(Phenylmethyl)	7.85	49.05	C14H16N2O2	244
20	9-Octadecenamide (Z)$$ Oleamide	1.92	49.23	C18H35NO	281

^a^*Rt* Retention time (minutes)

**Table 9:** Chemical composition of Khuz-2 strain using GC/MS analysis

No	Compound name	Percentage	Rt ^a^	Formula	Molecular weight
1	Pirrolidine, 1-[8-(3-octyloxiranyl)-1-oxooctyl]	24.17	11.654	C2H2Cl2O2	351
2	Pirrolidine, 1-[8-(3-octyloxiranyl)-1-oxooctyl]	1.26	18.249	C22H41NO2	351
3	2-Piperidinone$$ alpha-Piperidone	2.69	20.615	C22H41NO2	99
4	Undecane, 3,7-dimethyl-	1.11	25.451	C5H9NO	184
5	Undecane,2,4-dimethyl-	0.95	31.306	C13H28	184
6	n-nonadecane	1.26	31.6	C13H28	268
7	Hexadecane	0.79	36.999	C19H40	226
8	2-Decene,3-methyl-,(Z)	12.01	37.802	C16H34	154
9	Heptadecane 4-methyl	0.63	37.975	C11H22	254
10	Pyrrolo[1,2-a] pyrazine-1,4-dion,hexahydro-3-(2-methylpropyle)	39.9	38.921	C18H38	210
11	Nonadecane	0.63	41.807	C19H40	268
12	Ergotaman-3'.6'.18-trione, 9,10-dihydro-12'hydroxy-2'-methyl-5'-(phenylmethyl)-,(5'.alpha.,10 alpha)	1.90	48.197	C33H37N5O5	583
13	Pyrrolo[1,2-a] pyrazine-1,4-dion,hexahydro-3-(phenylmethyl)	10.74	49.033	C14H16N2O2	244
14	9-Octadecenamide (Z)$$ Oleamide	1.90	49.224	C18H35NO	281

^a^*Rt* Retention time (minutes)

**Table 10:** Chemical composition of Khuz-3 strain using GC/MS analysis

No	Compound name	Percentage	Rt ^a^	Formula	Molecular weight
1	Trans-2-hexenal	4.40	11.729	C6H10O	98
2	Pyrazin, 2,6-dimethyl	0.94	12.404	C6H8N2	108
3	Propan, 1,1-dichloro	0.37	13.891	C3H6Cl2	112
4	Dodecane	0.32	18.27	C12H26	170
5	2-Piperidone	2.51	21.226	C5H9NO	99
6	Dodecane-5-methyl	0.17	24.919	C13H28	184
7	Tetradecane	0.45	25.48	C14H30	198
8	Tetradecane	0.17	26.799	C14H30	198
9	Tetradecane	0.00		C15H32	212
10	1-dodecene	0.37	30.405	C12H24	168
11	1-Dodecanol	0.00		C12H26O	186
12	Phenol, 2,4-bis(1, 1-dimethylethyl)	0.52	31.312	C14H22O	206
13	Tetradecane, 4-ethyl-	0.57	31.631	C16H34	226
14	Tetradecane	0.25	32.756	C14H30	198
15	Tetradecane	0.30	34.083	C14H30	198
16	Hexadecane	0.51	36.516	C16H34	226
17	1- octanol, 2-butyl-	0.44	36.748	C12H26O	186
18	Pentacosane	0.89	37.025	C25H52	352
19	2- Decene, 3-methyl- (Z)	9.27	38.411	C11h22	154
20	Pyrrolo[1,2-a] pyrazine-1,4-dion,hexahydro-3-(2-methylpropyle)	1.03	38.747	C11H18N2O2	210
21	Pyrrolo[1,2-a] pyrazine-1,4-dion,hexahydro-3-(2-methylpropyle)	37.23	41.32	C11H18N2O2	210
22	Tetradecane	0.83	43.141	C14H30	198
23	Nonadecanamide	1.72	46.058	C19H39NO	297
24	Pyrrolidine, 1-(1,6-dooxooctadecyl)-	2.80	46.907	C22H41NO2	351
25	9-Octadecenamide (Z)$$ Oleamide	33.95	49.59	C18H35NO	281
26	Trans-2-hexenal	4.40	11.729	C6H10O	98
27	Pyrazin, 2,6-dimethyl	0.94	12.404	C6H8N2	108

^a^*Rt* Retention time (minutes)

Based on the results for strain khuz1, three compounds were detected in higher concentration compared to other found compounds including Pyrrolo[1,2-a] pyrazine-1,4-dion,hexahydro-3-(2-methylpropyle) (PPDHM) (20.51%), 1- Propanamine-2-methyl (13.30%), and 2- Decene, 3-methyl- (Z) (9.93%%). According to the results for strain khuz 2, four compounds were detected in higher concentration compared to other found compounds including Pyrrolo[1,2-a] pyrazine-1,4-dion, hexahydro-3-(2-methylpropyle) (39.9%), Pirrolidine, 1-[8-(3-octyloxiranyl)-1-oxooctyl] (24.1%), 2-Decene,3-methyl-,(Z) (12%), and Pyrrolo[1,2-a] pyrazine-1,4-dion,hexahydro-3-(phenylmethyl) (10.7%). In addition, for strain khuz 3, three compounds were detected in higher concentration compared to other found compounds including: Pyrrolo[1,2-a] pyrazine-1,4-dion,hexahydro-3-(2-methylpropyle) (37.23%), 9-Octadecenamide (Z)$$ Oleamide (33.9%), and 2- Decene, 3-methyl- (Z) (9.26%).

## Discussion

In recent decades, the incidence of resistance to antimicrobial agents has increased greatly due to the overuse of antibiotics worldwide [27]. Today, resistance to antimicrobial agents is one of the greatest health problems and threatens human health worldwide. Therefore, the findings of new antibiotics are an important point for the dissolving of the problems [2]. Recently, the researchers have found that a variety of natural sources, such as essential oil and extraction of herbs [24, 28, 29], environmental bacteria [23] and unhazardous fungi [30] can be used as novel antibiotics agents. Scientifics information on the safety and effectiveness of natural antibiotics’ remedies have shown that these agents have good anti-pathogenic activity with very low side effects [7, 8]. The among microorganisms, environmental bacteria have various beneficial potations in medical and pharmaceutical applications such as bio-absorption of heavy metals [31], bio-remediation of toxics [14], production of antimicrobial peptides and antibiotics [32–34]. One of the most important methods for strain identification and distribution is 16S rRNA sequencing method [11]. In this study, the results of 16S rRNA sequencing determined that the isolates belong to *Bacillus* sp. Isolated strains based on their high anti-fungal activity was closely related to *B. safensis* and *B. velezensis.* Several *Bacillus* have a suppressive effect on the growth of certain phytopathogens and could be used as biocontrol and bioremedial agents [12]. Some members of *Bacillus* genus have high potentials for usage as control agents against various fungal diseases [35]. In the current study, the sensitivity of *Bacillus* sp. was investigated for various used antibiotics. All isolated were highly resistant to Cefazolin and Cefalexin. Strain Khuz 3 did not show any sensitivity to Rafampin which is in contrast to *B. velezensis.* On the other hand, among the isolates, *B. safensis* strain Khuz1 did not show any inhibition zone. Strain Khuz 2 belonging to *B. rugosus* had antifungal and anti-bacterial effects against human and plant pathogen including *C. albicans, E. amylovera, R. radiobacter, F. axysparum, A. flavus, N. crassa* and *B. cinera.*

Considering the GC–MS results of three strains (Khuz1, Khuz 2, and Khuz 3), PPDHM was the most dominant chemical found in all strains. PPDHM is a heterocyclic compound belonging to diketopiperazine group, which has various pharmacological effects. In addition, chemicals of diketopiperazine group are produced as secondary metabolites by microorganisms with a wide range of bioactivity [36]. The compounds of diketopiperazine group are one of the smallest classes of cyclic peptides with various pharmaceutical effects such as antimicrobial, antiviral, and antitumor activities [36]. Kiran et al., 2018 showed that pyrrolo[1,2-a]pyrazine-1,4-dione,hexahydro isolated from *Bacillus tequilensis* strain MSI45 could effectively control multi-drug resistant *Staphylococcus aureus* [37]. Furthermore, pyrrolo[1,2-a]pyrazine-1,4-dione, hexahydro from sponge associated *Bacillus* sp. was reported to reduce oxidative damage by radicals [38]. PPDHM was currently identified as a major chemical with antimicrobial effects in the metabolites of an endophytic *Mortierella alpina* strain isolated from the Antarctic moss *Schistidium antarctici* [39]. This compound was also previously isolated from sponge-associated bacteria and showed bioactivity including antibiofilm and antilarval activity against *Vibrio halioticoli* [40].

**Fig. 3:**
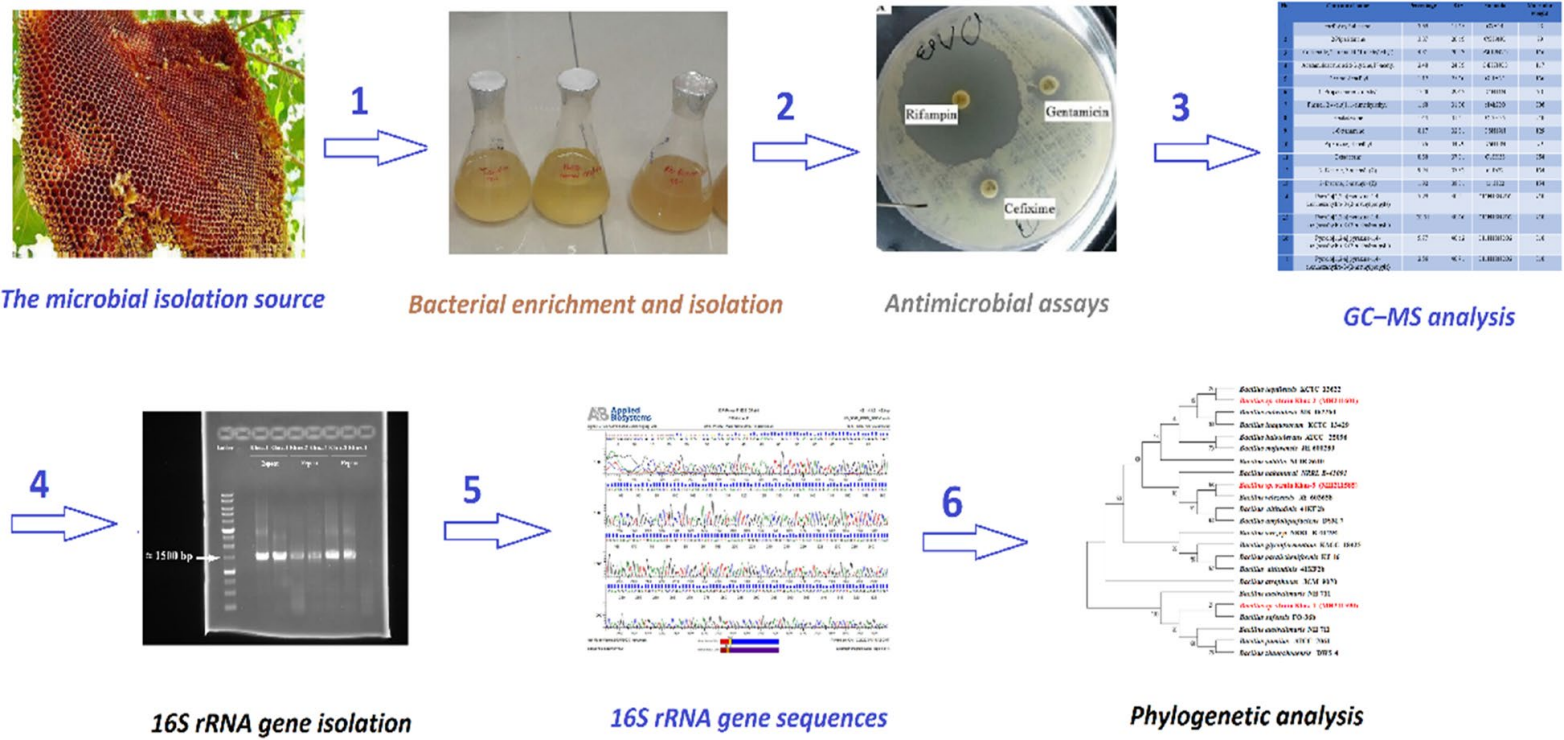
A schematic flow chart of all the procedures

The other most dominant chemical found in Khuz 2 which belongs to diketopiperazine group was Pyrrolo [1,2-a] pyrazine-1,4-dion, hexahydro-3-(phenylmethyl) (PPDHP). Sanjenbam and coworkers reported anticandidal activities of PPDHP from *Streptomyces* sp. VITPK9 (isolated from a brine spring soil sediment sample) against *Candida albicans, Candida krusei,* and *Candida tropicalis* [22]. In addition, the bioactivity of PPDHP has been investigated by [41]. Based on the results, PPDHP was less toxic to the normal cell and is nonhemolytic at low concentrations of < 100 μg/mL. In addition, genotoxicity study showed that PPDHP had minimal chromosomal aberrations relative to streptomycin. On the whole, the GC–MS analysis showed the presence of potential antimicrobial agents belonging to diketopiperazine group PPDHM and PPDHP as the major compounds. Therefore, *Bacillus* sp. which was isolated from wild honey is a promising bacteria for the biotechnological production of antibiotics. All the procedures are summarized in Fig. 3 as a schematic flow chart.

## Conclusion

It has been considered the critical lack of new antimicrobials is the clinical pipeline. In 2019 WHO identified only six innovative antibiotics, which were used in the clinical development of priority pathogens. However, a lack of access to quality antimicrobials remained a major issue. We have isolated three strain of *Bacillus* genus from honey that differs in their antagonistic activity against a number from phytopathogenic and human fungi. Only *B. rugosus* strain Khuz 2 was able to protect *C. albicans* human fungi. Thus, *B. rugosus* stain Khuz 2 can find application as antibacterial and antifungus in agriculture as bioinoculant for agriculturally important plants. The major compounds of strain Khuz 1, 2 and 3 obtained from GC–MS are Pyrrolo[1,2-a] pyrazine-1,4-dion, hexahydro-3-(2-methylpropyl).

## Supplementary Information

**Additional file 1.**

**Additional file 2.**

**Additional file 3.**

## Data Availability

The datasets used and/or analyzed during the current study are available from the corresponding author on reasonable request.

## Abbreviations

GC–MS: Gas chromatography–mass spectrometry
PPDHM: Pyrrolo [1,2-a] pyrazine-1,4-dion, hexahydro-3-(2-methylpropyle)
Khuz 1: Khuzestan1
Khuz 2: Khuzestan2
Khuz 3: Khuzestan3
DNA: Deoxyribonucleic Acid
OD: Optical Density
MIC: Minimum inhibitory concentration
MBC: Minimum bactericidal concentration
