# Supplementary figures and images for: Antimicrobial activity of Bacillus sp. isolated strains of wild honey
Source: BMC Complement Med Ther. 2022 Mar 19;22:78. doi: 10.1186/s12906-022-03551-y (PMC8933914; doi:10.1186/s12906-022-03551-y)

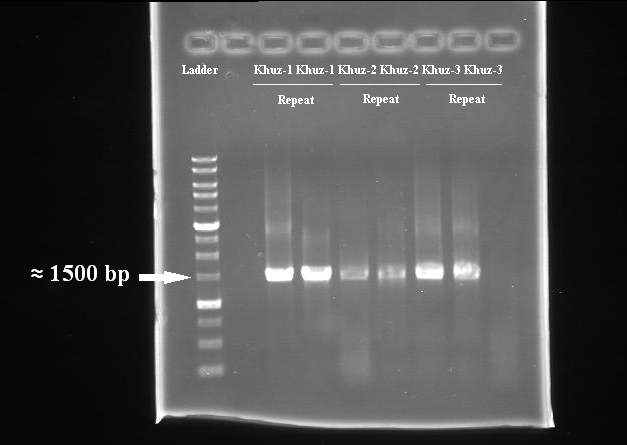

Supplement: Supplementary file 1 — Additional file 1. [file 12906_2022_3551_MOESM1_ESM.png]

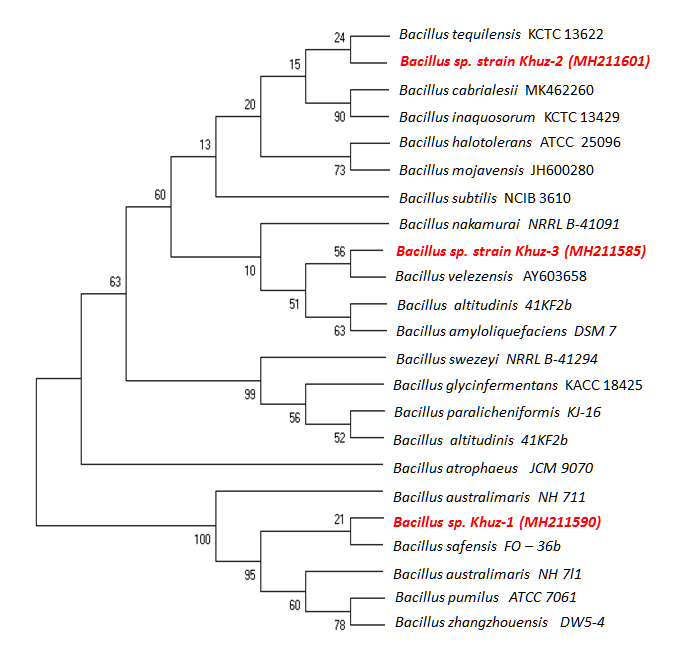

Supplement: Supplementary file 2 — Additional file 2. [file 12906_2022_3551_MOESM2_ESM.png]

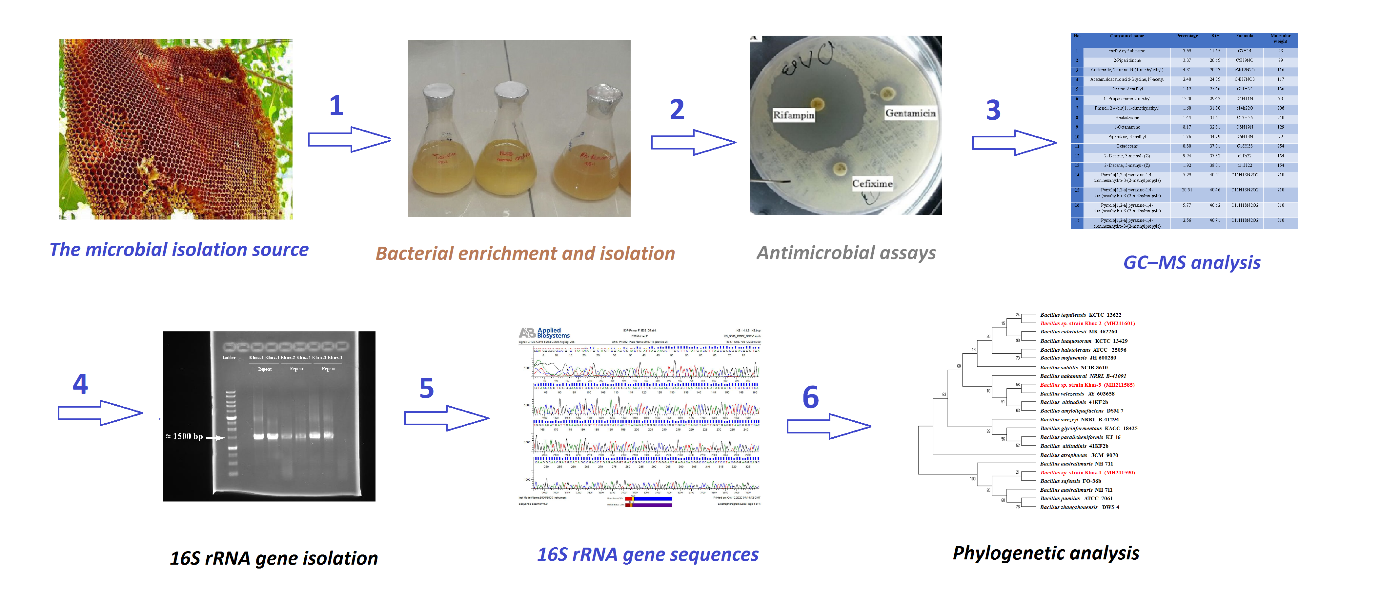

Supplement: Supplementary file 3 — Additional file 3. [file 12906_2022_3551_MOESM3_ESM.png]
